# Mechanism of auranofin toxicity: Inhibition of tetrahydrobiopterin metabolism

**DOI:** 10.1016/j.taap.2025.117615

**Published:** 2025-10-25

**Authors:** Shaojun Yang, Yi-Hua Jan, Jason R. Richardson, Diane E. Heck, Jeffrey D. Laskin

**Affiliations:** a Department of Environmental and Occupational Health and Justice, Rutgers University School of Public Health, Piscataway, NJ, USA; b Department of Physiology and Pharmacology, College of Veterinary Medicine, University of Georgia, Athens, GA, USA; c Department of Pharmacology and Toxicology, Ernest Mario School of Pharmacy, Rutgers University, Piscataway, NJ, USA

**Keywords:** Tetrahydrobiopterin, Rheumatoid, Arthritis, Methotrexate, Sepiapterin, Folic Acid

## Abstract

Auranofin is a gold-containing anti-inflammatory drug used for the treatment of rheumatoid arthritis. Recent studies indicate that auranofin targets cellular antioxidants including the thioredoxin system and the ubiquitin–proteasome system, which can result in cellular oxidative stress. In the present studies, we identified a novel mechanistic site of action of auranofin, namely tetrahydrobiopterin (BH4) biosynthesis. BH4 is an essential cofactor required for aromatic amino acid hydroxylases, nitric oxide synthase and alkylglycerol monooxygenase, enzymes that produce mediators important in regulating inflammation. It is synthesized *de novo* from GTP via the action of GTP cyclohydrolase I, 6-pyruvoyl-tetrahydrobiopterin synthase and sepiapterin reductase (SPR), which leads to the production of dihydrobiopterin (BH2). Further metabolism of BH2 to BH4 is mediated by dihydrofolate reductase (DHFR). We discovered that auranofin causes a rapid depletion of cellular BH4 in several different cell types; PC12 cells were most sensitive to auranofin followed by Jurkat cells, BeWo cells, HaCaT cells, SKNMC cells, RAW246.7 cells and CX-1 cells. This was due to the inhibition of both SPR and DHFR. BH4 is required for tyrosine hydroxylase and tryptophan hydroxylase, enzymes mediating the synthesis of dopamine and serotonin, respectively. In PC12 cells, blocking BH4 biosynthesis with auranofin correlated directly with inhibition of serotonin and dopamine production indicating that the drug targeted aromatic amino acid hydroxylase activity. Our findings provide novel insights into the metabolic consequences of treating inflammatory diseases or cancer cell growth and metastasis with auranofin.

## 1. Introduction

**Fig. 1.**
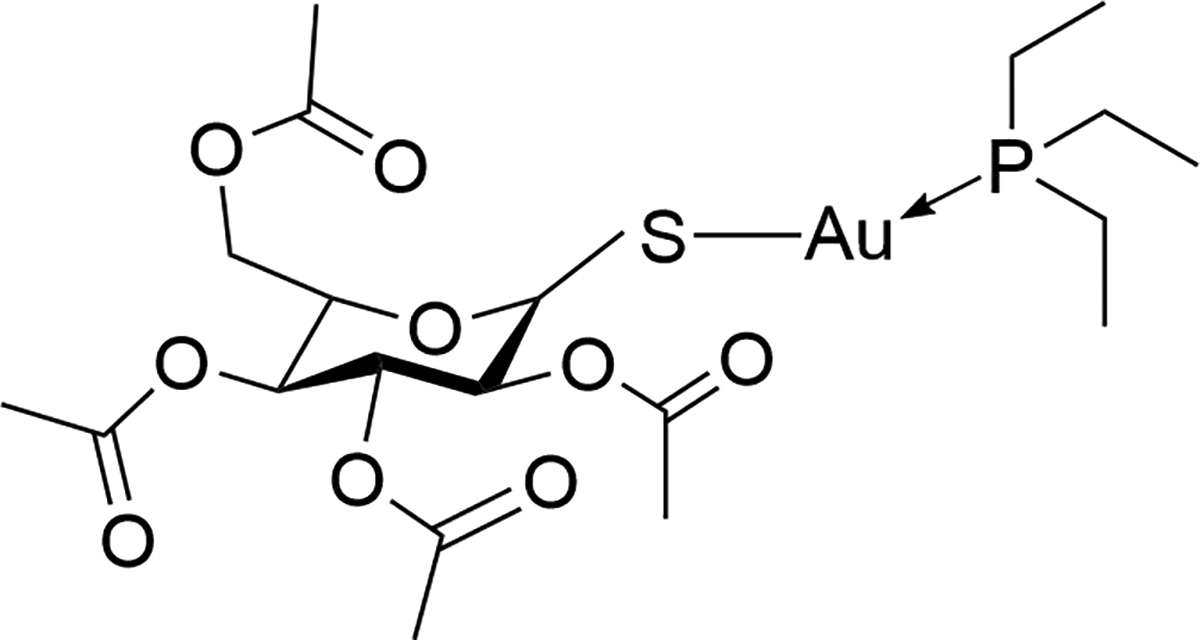
Structure of auranofin. Auranofin is a gold(I) containing compound coordinated to a tetraacetyl thioglucose ligand and a triethylphosphine ligand. The gold(I) triethylphosphine ligand is thought to be the most active component of the drug.

Auranofin (2,3,4,6-tetra-*O*-acetyl-1-thio-β-D-glucopyranosato-*S*-triethyl phosphine gold, see Fig. 1 for structure) is an anti-inflammatory drug developed for the treatment of rheumatoid arthritis (Finkelstein et al., 1976; Kean et al., 1997; Mertens et al., 2023). Over the past several years, auranofin has been investigated as a therapeuitc for other medical conditions including microbial and parasitic infections, neurodegenerative disorders and cancer (Nobili et al., 2010; Madeira et al., 2015; Onodera et al., 2019; Arguello-Garcia et al., 2020; Abdalbari and Telleria, 2021; Shen et al., 2023; Coscione et al., 2024; Chen et al., 2025). The effects of auranofin on cancer are particularly noteworthy as significant antitumor activity has been demonstrated not only in animal models of chronic lymphocytic leukemia, osteosarcoma and colon, breast and lung cancer (Fiskus et al., 2014; Li et al., 2016; Afrifa et al., 2024; Kinoshita et al., 2024; Zhang et al., 2024), but also in early clinical trials for ovarian cancer, lung cancer and chronic lymphocytic leukemia (NCT01419691; NCT01737502; NCT01747798; NCT03456700). The precise mechanism of action of auranofin is not known; the drug has a high affinity for cysteine, selenocysteine, lysine and histidine amino acid residues in proteins (Dos Santos, 2014) and has been reported to inhibit thioredoxin reductase, a selenocysteine-containing enzyme important in redox homeostasis (Gromer et al., 1998; Bindoli et al., 2009; Gencheva and Arner, 2022; Gencheva et al., 2022). More recently, auranofin has been reported to interact with thiol pairs in proteins such as thioredoxin, a reaction known to result in the formation of gold disulfide adducts (Quadros Barse et al., 2025). Auranofin also modifies the ubiquitin–proteasome system, resulting in inhibition of proteasome-associated deubiquitinase or increases in enzymes that generate activated ubiquitin, a molecule important in the regulation of protein homeostasis (Liu et al., 2014; Yan et al., 2023). By inhibiting the thioredoxin-thioredoxin reductase system and/or modifying the ubiquitin–proteasome system, auranofin causes oxidative stress, a process that can lead to apoptosis and cell death (Shen et al., 2023; Castro et al., 2025).

**Fig. 2.**
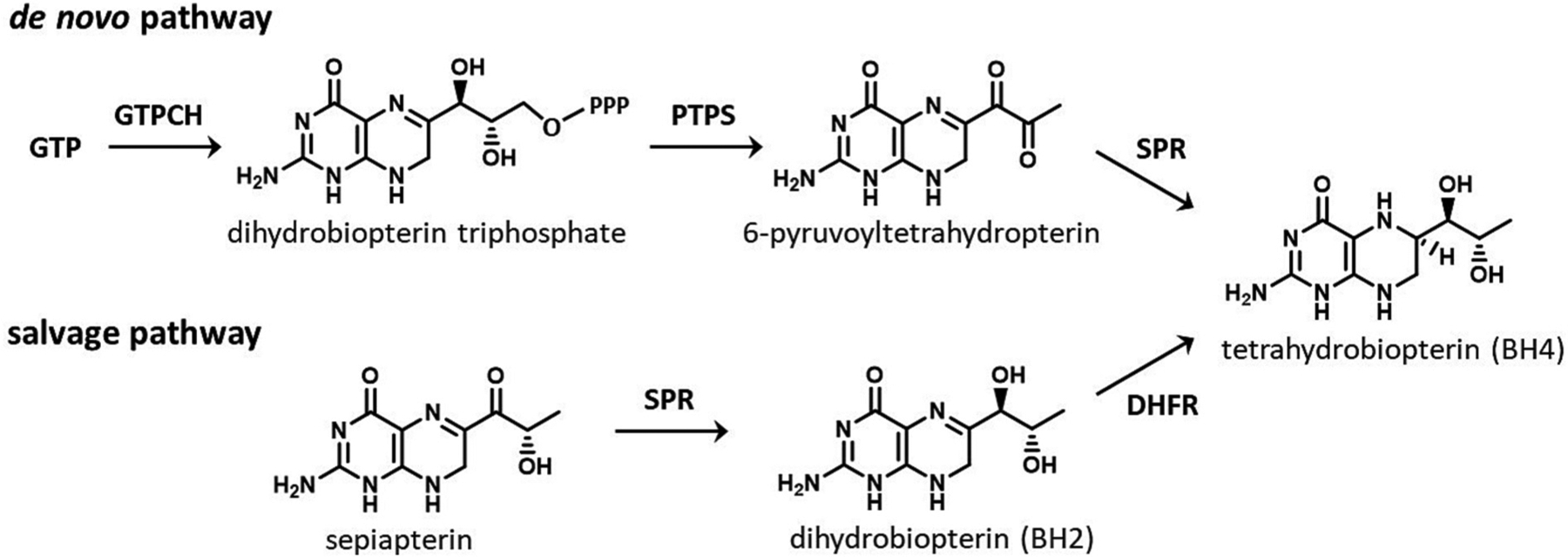
*De novo* and salvage pathways for tetrahydrobiopterin (BH4) biosynthesis. Synthesis of BH4 *de novo* is initiated by the metabolism of GTP to dihydroneopterin triphosphate via GTP cyclohydrolase I (GTPCH) followed by its metabolism to 6-pyruvoyltetrahydrobiopterin (PTP) by PTP synthase (PTPS). Sepiapterin reductase (SPR) catalyzes the metabolism of PTP to BH4. Sepiapterin is generated non-enzymatically from PTP. In the salvage pathway, SPR catalyzes the reduction of sepiapterin to BH2; dihydrofolic acid reductase (DHFR) mediates metabolism of BH2 to BH4.

Sepiapterin reductase (SPR, 7,8-dihydrobiopterin:NADP+ oxidoreductase) is an NADPH-dependent enzyme that catalyzes the formation of dihydrobiopterin (BH2) from sepiapterin (Yang et al., 2013; Yang et al., 2015), which is synthesized *de novo* from guanosine triphosphate (GTP) via GTP cyclohydrolase I (GTPCH) and 6-pyruvoyl-tetrahydrobiopterin synthase (PTPS) (see Fig. 2 for metabolic pathway). BH2 is metabolized by dihydrofolate reductase (DHFR) to tetrahydrobiopterin (BH4), an essential cofactor for nitric oxide synthases, alkylglycerol monooxygenase (AGMO), and aromatic amino acid hydroxylases (Werner et al., 2011), enzymes linked to inflammation and cancer (Flierl et al., 2008; Vedenko et al., 2020; Sailer et al., 2021; Clark et al., 2023; Ahangari and Norioun, 2025). For example, BH4 controls electron flow through the nitric oxide synthase reaction cycle, which generates nitric oxide, a proinflammatory mediator (Korhonen et al., 2005; Tejero and Stuehr, 2013; Garcia and Sessa, 2019; Iyanagi, 2019; Jiang et al., 2025). AGMO is a mixed function oxidase that degrades ether lipids (Magnusson and Haraldsson, 2011; Watschinger and Werner, 2013; Dorninger et al., 2020). In cancer cells, this can promote tumor progression (Sailer et al., 2021); ether lipids can also act as immune signaling molecules, promoting inflammation (Magnusson and Haraldsson, 2011; Watschinger and Werner, 2013; Dorninger et al., 2020; Sailer et al., 2021). Aromatic amino acid hydroxylases are important in generating catecholamines such as dopamine, norepinephrine and epinephrine. In addition to their activity as neurotransmitters, they can stimulate the release of pro-inflammatory cytokines (Flierl et al., 2008). They have also been reported to enhance angiogenesis and tumor progression (Xiao et al., 2023; Ahangari and Norioun, 2025).

Current studies demonstrate that auranofin effectively inhibits the biosynthesis of both BH2 and BH4 across a range of cell types. These results suggest that auranofin exerts its pharmacological activity through multiple intracellular targets, modulating not only redox balance, but also signaling molecules regulating inflammation and cell proliferation. These findings suggest that agents that suppress BH2 and BH4 metabolism may offer therapeutic benefits not only in the treatment of inflammatory diseases such as arthritis, but also for suppression of tumor cell growth and metastasis.

## 2. Materials and methods

### 2.1. Chemicals

BH2 and BH4 were from Santa Cruz Biotechnology (Dallas, TX). Ham’s F-12 K (Kaighn’s), DMEM, fetal bovine serum and penicillin/streptomycin were from Thermo Fisher (Waltham, MA). Sepiapterin, horseradish peroxidase, NADPH, menadione, auranofin, methotrexate, NADPH, recombinant human DHFR, protease inhibitor mixture 1 and all other chemicals and enzymes were from Sigma-Aldrich (St. Louis, MO), unless otherwise indicated. Protease inhibitor mixture 1 contains 4-(2-aminoethyl) benzenesulfonyl fluoride, pepstatin A, E-64 [transepoxysuccinyl-l-leucylamido-(4-guanidino) butane], bestatin, leu-peptin, and aprotinin. Human recombinant SPR was prepared in our laboratory as previously described (Yang et al., 2013; Yang et al., 2015).

### 2.2. Cells

HaCaT, PC12, CX-1, SKNMC, RAW264.7 and Jurkat cells were cultured in DMEM; BeWo cells were cultured in Ham’s F-12 K medium. All culture medium was supplemented with penicillin (100 IU/ml), streptomycin (100 μg/ml) and 10 % fetal bovine serum. Cells were cultured in a humidified atmosphere of 5 % CO_2_ at 37 °C. Cell viability was determined using alamarBlue (BioSource International, Camarillo, CA) as previously described (Jan et al., 2010).

### 2.3. Enzyme assays

Sepiapterin reductase activity was assayed by measuring decreases in sepiapterin absorbance at 420 nm as described by Kato (Kato, 1971) with some modifications. Briefly, standard reaction mixes contained 100 mM potassium phosphate buffer, pH 7.0, 100 μM NADPH, 50 μM sepiapterin and 1 μg of enzyme protein in a final volume of 0.2 ml. Changes in absorbance of sepiapterin were monitored using a Spectramax M5 microplate reader (Molecular Devices, Sunnyvale, CA). NADPH, menadione and cofactors had minimal absorbance at 420 nm and did not interfere with the sepiapterin reductase assay. Chemical redox cycling by SPR was assayed by measuring the formation of superoxide anion in the presence of menadione as previously described (Yang et al., 2013). Reaction mixes contained 100 mM potassium phosphate buffer, pH 7.8, 50 μM acetylated cytochrome *c*, 200 μM NADPH, 200 μM menadione and 1 μg of sepiapterin reductase in a final volume of 100 μl. Superoxide anion was measured spectrophotometrically by the reduction of acetylated cytochrome *c* at 550 nm. DHFR activity was measured as previously described (Whitsett et al., 2013) with some modifications. Standard reaction mixes contained 100 mM potassium phosphate buffer, pH 7.0, 200 μM NADPH, 50 μM BH2 and 5 μg recombinant human DHFR in a final volume of 0.2 ml. Enzyme activity was monitored by decreases in absorbance of NADPH at 340 nm using a microplate reader. Auranofin or PBS control was added to reaction mixes 10 min prior to initiating enzyme assays by the addition of NADPH.

### 2.4. Assays for cellular BH2 and BH4

Metabolic products of sepiapterin (BH2 and BH4) in intact cells and cell lysates were measured using HPLC as previously described (Ferre and Naylor, 1988) with some modifications (Yang et al., 2013; Yang et al., 2015). Briefly, cells in six-well culture dishes were grown until 60–70 % confluent (~1 × 10^6^ cells/well). The growth medium was then removed and the cells incubated with growth medium containing 100 μM sepiapterin. After 3 h, cells were harvested, centrifuged (1500 *g*, 10 min), and pellets suspended in 200 μl of lysis buffer (50 mM Tris-HCl, pH 7.4, 137 mM NaCl, 0.5 % Nonidet P-40 (NP-40), 0.5 mM EDTA) for 20 min on ice. Following centrifugation (20,000 *g*, 20 min), 25 μl of 1 M NaOH and 25 μl of a mixture of 80 mM iodine and 120 mM KI in 1 M HCl was added to the supernatants. Thirty min later, 50 μl of 1 M H_3_PO_4_ was added to the supernatants and the precipitated proteins removed by centrifugation (15,000 *g*, 3 min). After standing for 10 min at room temperature, excess iodine was reduced by the addition of 25 μl of 57 mM ascorbic acid to the reaction mix. BH2 and BH4 were then analyzed on a Jasco high-performance liquid chromatography system (Easton, MD) fitted with a Maxsil-10 C18 column (250 × 4 mm; Phenomenex, Torrance, CA). Fluorescence was monitored using a Jasco FP-2020 spectrofluorometer with excitation and emission wavelengths set at 362 and 435 nm, respectively (Yang et al., 2013; Yang et al., 2015). In inhibitor experiments, cells were pretreated with auranofin and/or methotrexate for 2 h prior to the addition of sepiapterin. Cell viability following drug treatments was greater than 95 %.

### 2.5. Assay for catecholamine/monoamines

Catecholamine and monoamine production in PC12 cells were assayed as previously described (Yang et al., 2015). Briefly, cells grown in 10-cm culture dishes were treated in the absence or presence of auranofin (0.04–2.5 μM) in 10 ml of growth medium without FBS. After 24 h, the cells were removed from the plates, washed with phosphate-buffered saline, and centrifuged (1500 *g*, 10 min). Cell pellets (~8 × 10^6^ cells) were resuspended in 1 ml of 0.1 N perchloric acid containing 200 μM EDTA and 350 μM sodium metabisulfite and sonicated on ice. Ly-sates were centrifuged (20,000 *g*, 10 min) and clear supernatants filtered using 0.22-μm filters. Extracts were then analyzed for neurotransmitters and their metabolites using a Waters 2695 high-performance liquid chromatography system (Waters Corp., Milford, MA) fitted with a cation exchange column (MD-150 3.2 × 15 mm column; ESA Biosciences, Inc., Chelmsford, MA) using an isocratic mobile phase (MD-TM mobile phase; ESA Biosciences Inc.) containing 2.2 mM NaCl pumped at constant flow rate of 0.5 ml/min. Compounds were quantified by electrochemical detection using a glassy carbon working electrode (2 mm in diameter) with an in situ silver reference electrode (flow cell, 2 mm GC WE, ISAAC; Waters Corp.) (Yochum et al., 2014).

## 3. Results

### 3.1. Effects of auranofin on SPR activity

**Fig. 3.**
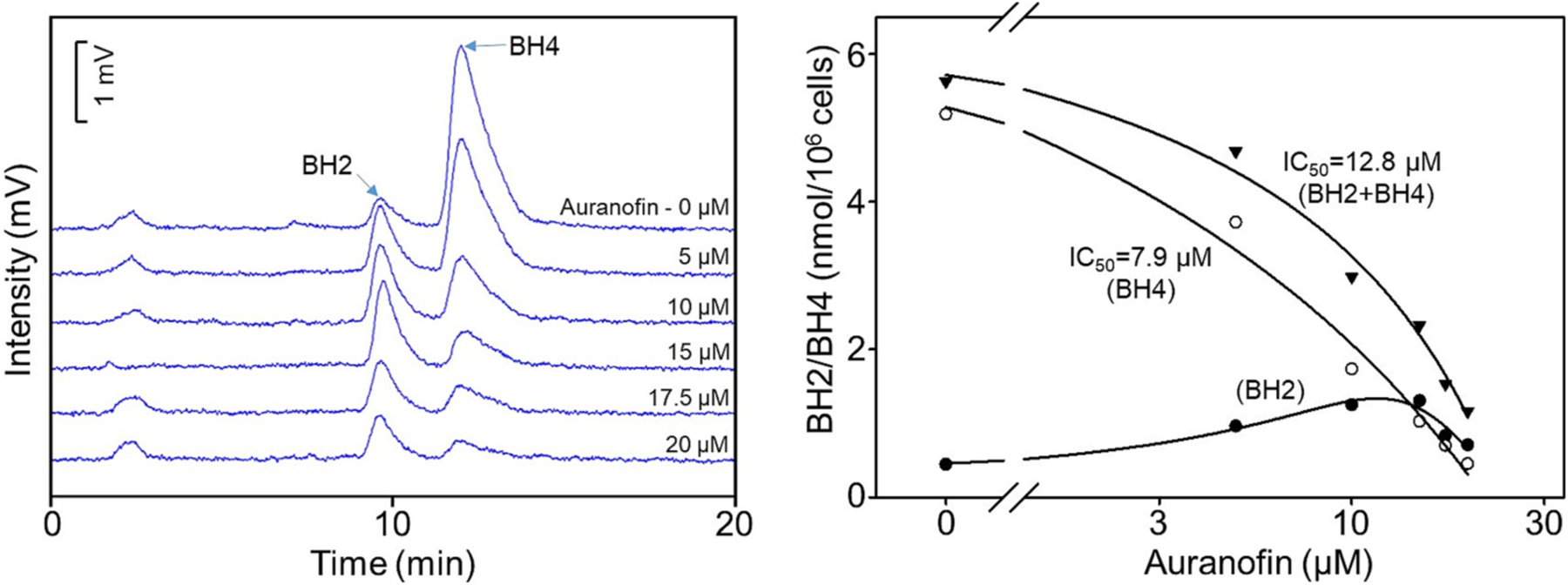
Effects of auranofin on BH2 and BH4 production in intact HaCaT cells treated with sepiapterin. HaCaT cells (~1 × 10^6^ cells/well in 6 well culture plates) were incubated with control medium or medium containing increasing concentrations of auranofin for 2 h followed by 100 μM sepiapterin. After an additional 3 h, BH2 and BH4 were extracted from the cells and analyzed by HPLC with fluorescence detection. *Left panel*: Chromatograms showing BH2 and BH4 content of control cells and cells treated with Auranofin. *Right panel*: Concentration-dependent inhibition of BH2 and BH4 formation in cells treated with auranofin.

**Fig. 4.**
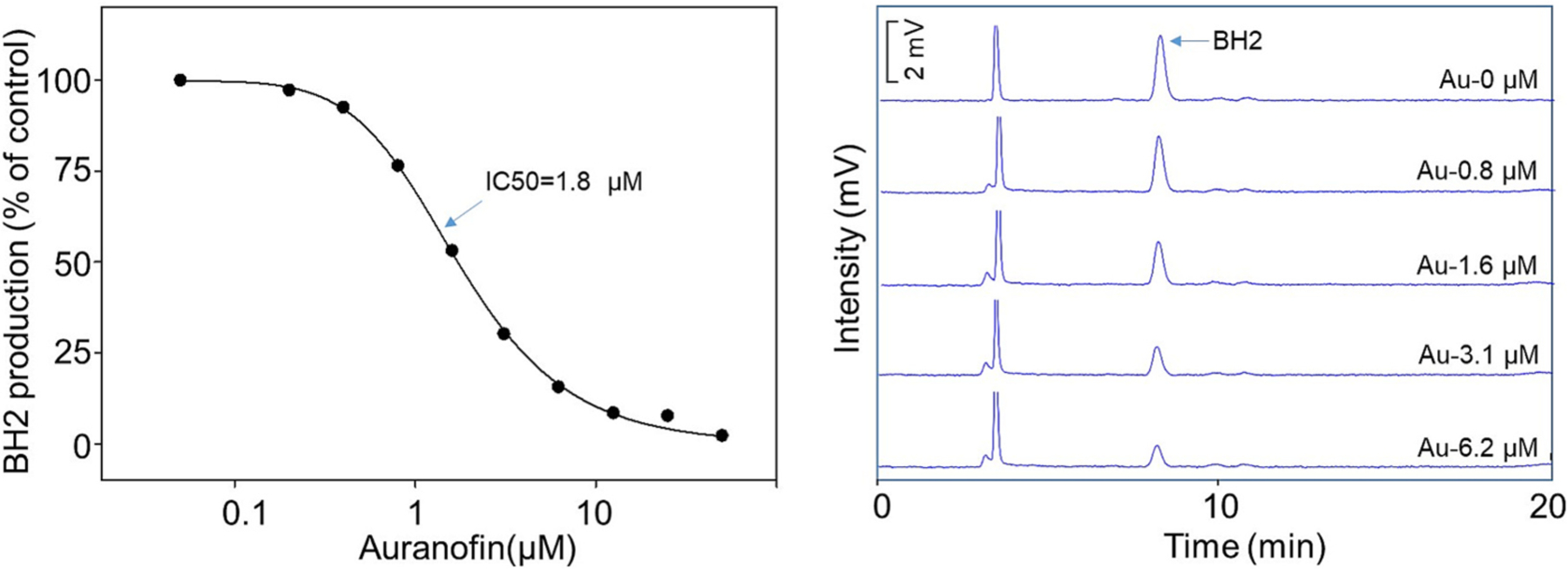
Effects of auranofin on sepiapterin reductase (SPR) enzyme activity in HaCaT lysates. Lysates were assayed for SPR enzyme activity in control reaction mixes or reaction mixes containing increasing concentrations of auranofin (left panel). SPR activity was determined by decreases in BH2 formation from sepiapterin as measured by HPLC (right panel).

**Fig. 5.**
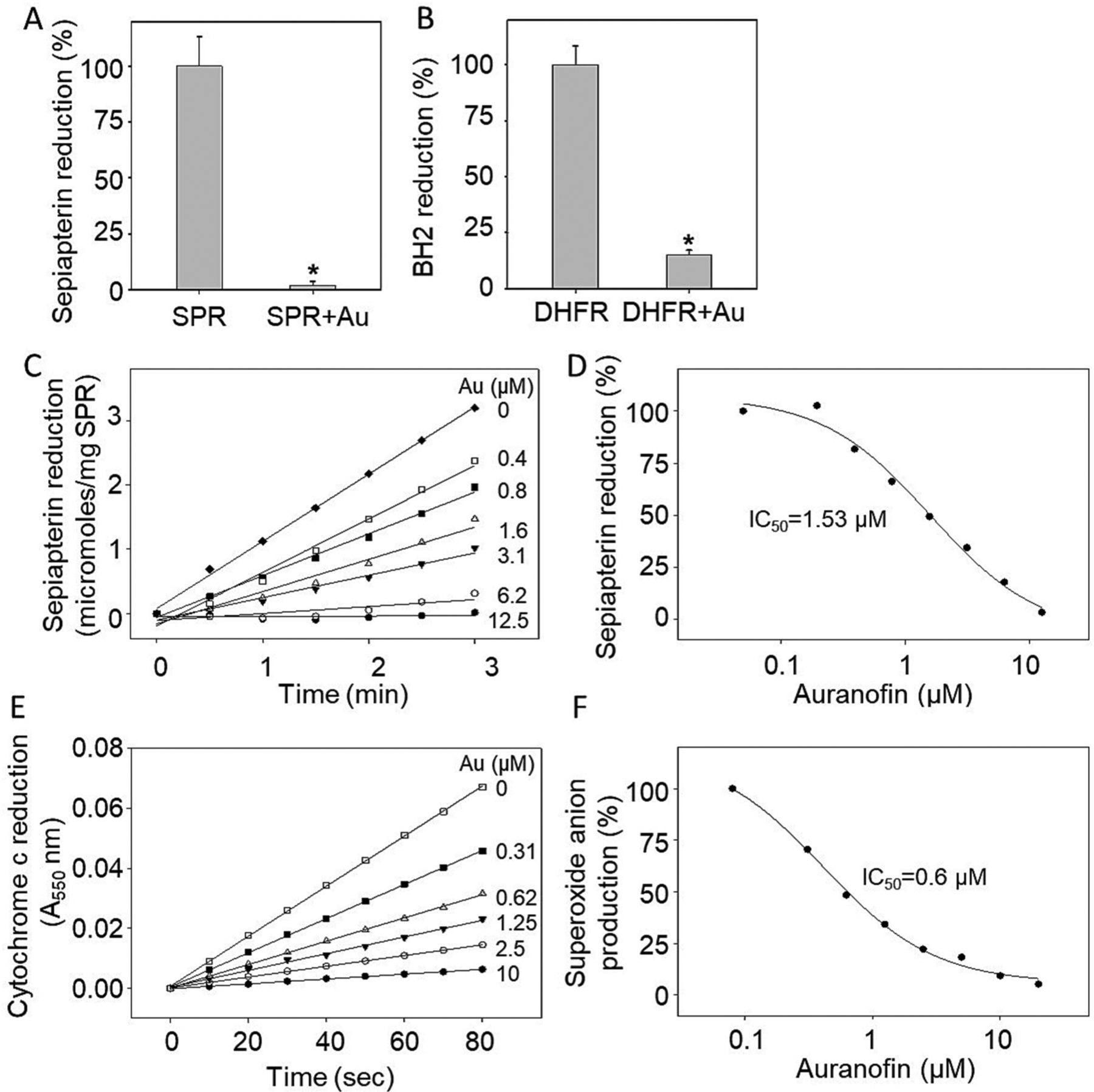
Effects of auranofin on human recombinant sepiapterin reductase (SPR) and dihydrofolate reductase (DHFR) activity. *Upper panels*. Enzyme assays with recombinant SPR and DHFR were performed with control reaction mixes or reaction mixes containing 10 μM auranofin (Au) (panels A and B). SPR was measured in enzyme assays by decreases in sepiapterin absorbance at 420 nm as described in the Materials and Methods. DHFR activity in enzyme assays was measured by decreases in absorbance of NADPH at 340 nm. Bars, mean ± SE (*n* = 3); *Significantly different (*P* < 0.05) from control samples in the absence of auranofin. *Middle panels*. Effects of increasing concentrations of auranofin on sepiapterin reduction by recombinant SPR (panels C and D). *Lower panels*. Effects of increasing concentrations of auranofin on quinone redox cycling by recombinant SPR (panels E and F). Redox cycling was measured spectrophotometrically by the reduction of acetylated cytochrome *c* at 550 nm during the formation of superoxide anion in enzyme assays in the presence of 100 μM menadione.

In initial studies, the effects of auranofin on SPR in HaCaT cells were analyzed. These cells were found to readily take up and utilize sepiapterin, where it is metabolized to BH2 and BH4. These data indicate that the cells contain SPR enzyme activity (Fig. 3). SPR enzyme activity was confirmed in HaCaT cell lysates; enzyme activity was dependent on NADPH (Fig. 4 and not shown). In intact HaCaT cells and HaCaT cell lysates, auranofin caused a concentration-dependent inhibition SPR (IC50 = 12.8 μM and 1.8 μM, respectively) (Figs. 3 and 4). In further studies, we used recombinant human SPR to evaluate the effect of auranofin on the enzyme. Auranofin caused a concentration-dependent inhibition of sepiapterin utilization by the recombinant enzyme (IC50 = 1.5 μM) (Fig. 5). In earlier studies, we demonstrated that SPR also mediates distinct NADPH-dependent chemical redox cycling activity (Yang et al., 2013). In the redox cycling process, one electron reduction of redox-active chemicals such as menadione (methyl-1,4-naphthoquinone) by SPR generates radical ions; these rapidly react with oxygen generating superoxide anion and the parent compound. Auranofin caused a concentration-dependent inhibition of chemical redox cycling, as measured by the formation of superoxide in enzyme assays (IC50 = 0.6 μM) (Fig. 5).

**Fig. 6.**
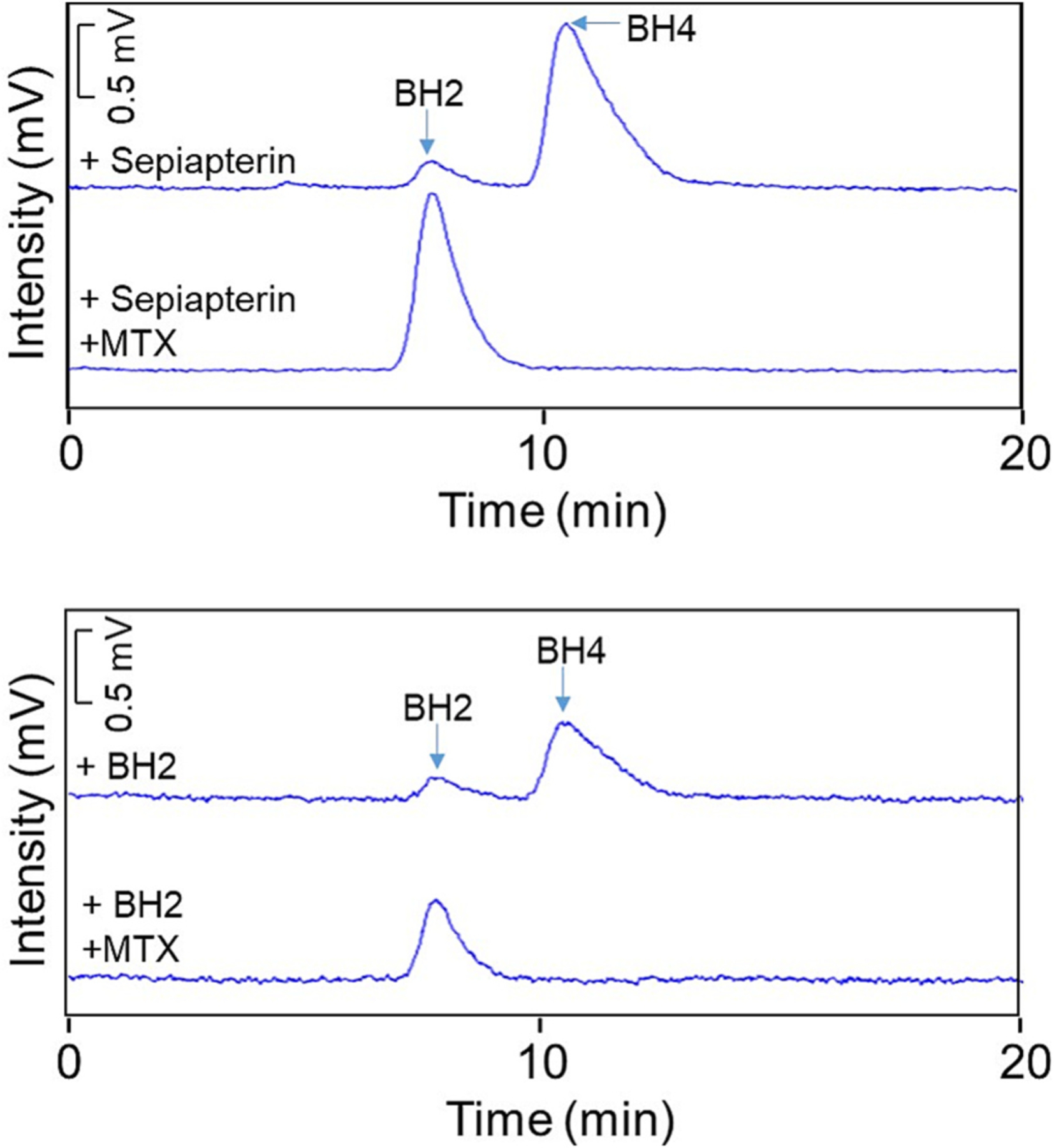
Effects of methotrexate on BH2 and BH4 metabolism in HaCaT cells. Cells were treated with control medium or medium containing MTX (1 μM, 2 h), an inhibitor of DHFR, followed by 200 μM sepiapterin (*upper panel*) or BH2 (*lower panel*). After an additional 3 h incubation, cells were rinsed with PBS; BH2 and BH4 were then extracted and analyzed by HPLC as described in the Materials and Methods.

**Fig. 7.**
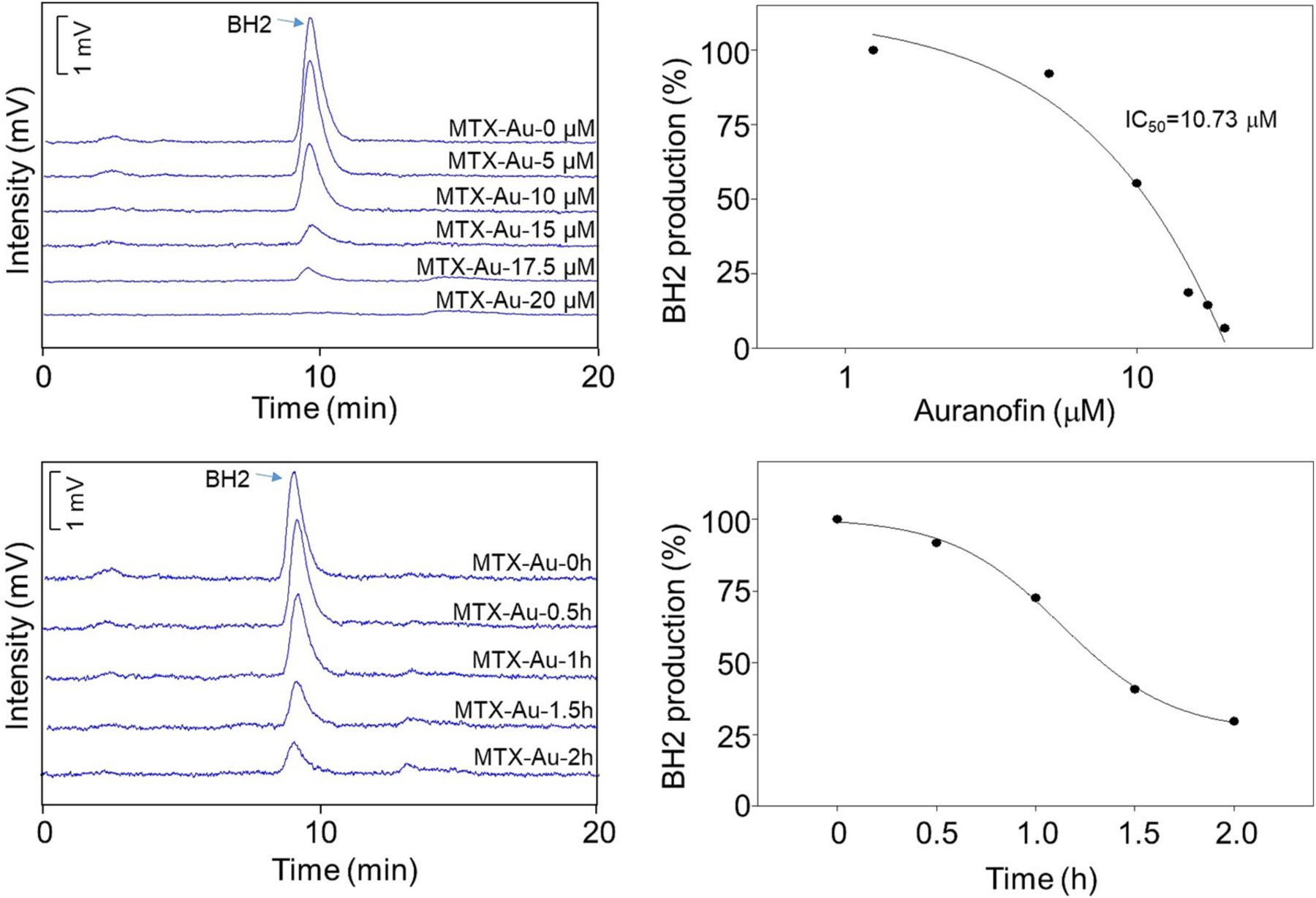
Effects of auranofin on sepiapterin reductase (SPR) activity in intact HaCaT cells. SPR enzyme activity was assayed by the metabolism of sepiapterin to BH2 in the presence of MTX (1 μM), which blocks metabolism of BH2 to BH4. Cells were pretreated with MTX for 2 h. *Upper panels*. HaCaT cells were treated with control medium or medium containing increasing concentrations of auranofin for 2 h followed by 200 μM sepiapterin. After an additional 3 h, cells were rinsed with PBS and BH2 analyzed by HPLC as described in the Materials and Methods. *Lower panels*. HaCaT cells were treated with auranofin for increasing periods of time (0–2*h*) followed by the addition of 200 μM sepiapterin. After an additional 3 h, cells were rinsed with PBS and analyzed for BH2 content.

SPR activity in HaCaT cells is assessed by the formation of BH2 and BH4 following treatment with sepiapterin. The metabolism of BH4 from BH2 is mediated by DHFR (Figs. 2 and 6). To distinguish between SPR and DHFR activity, cells were treated with methotrexate, a DHFR inhibitor. Methotrexate was found to inhibit the formation of BH4 from sepiapterin and resulting in BH2 accumulation in HaCaT cells. These data demonstrate that SPR activity can be assayed in cells independent of DHFR activity (Fig. 6). In HaCaT cells treated with methotrexate, auranofin was found to inhibit the formation of BH2, indicating that it directly inhibits SPR activity; the effects of auranofin were time-dependent with a maximum inhibition after 2 h at 20 μM (Fig. 7) indicating that the drug must be taken up by the cells and accumulate in sufficient concentrations to inhibit SPR. The IC50 for inhibiting cellular SPR by auranofin was 10.7 μM (Fig. 7).

**Fig. 8.**
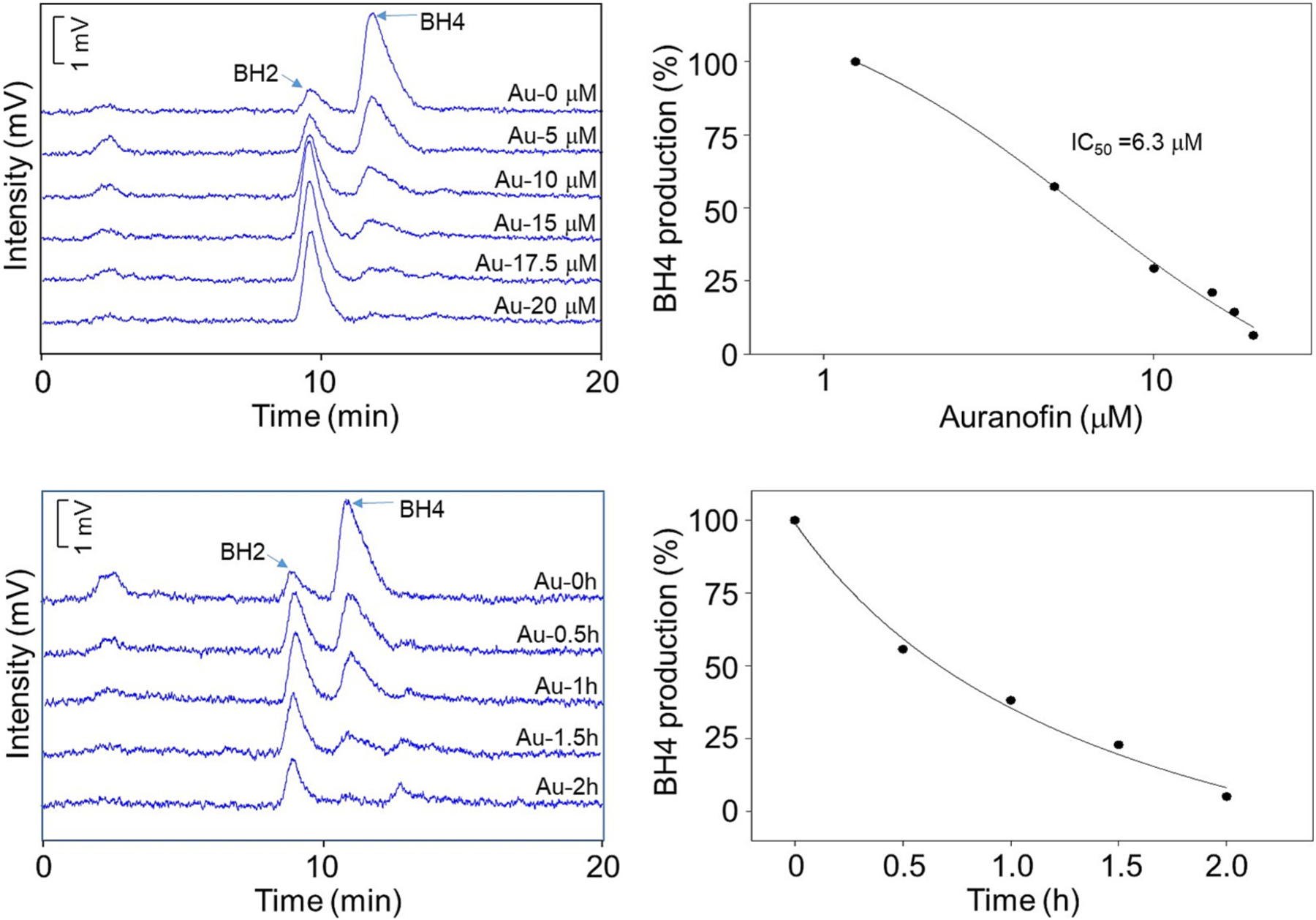
Effects of auranofin on dihydrofolate reductase (DHFR) activity in intact HaCaT cells. DHFR enzyme activity in the cells was assayed by the metabolism of BH2 to BH4. *Upper panels*. HaCaT cells were treated with control medium or medium containing increasing concentrations of auranofin for 2 h followed by the addition of 200 μM BH2. After an additional 3 h, cells were rinsed with PBS and BH2 and BH4 analyzed by HPLC as described in the Materials and Methods. *Lower panels*. Time-dependent inhibition of DHFR by auranofin. Cells were treated with the drug for increasing periods of time (0–2 h) followed by 200 μM BH2. After an additional 3 h, cells were rinsed with PBS and analyzed for BH2 and BH4 content.

In studies assessing the effects of auranofin on sepiapterin metabolism in HaCaT cells, we noted that increasing concentrations of the drug were correlated with increases in BH2 formation. This was associated with a corresponding decrease in the formation of BH4 (Fig. 3). These data suggest that auranofin also inhibits DHFR, the second step in the formation of BH4 from sepiapterin (Fig. 2). This was confirmed in human recombinant DHFR (Fig. 5). DHFR can also be directly measured in HaCaT cells by treatment with BH2, which is metabolized by the enzyme to BH4 (Fig. 6). Treatment of the cells with methotrexate prevented the metabolism of BH2 to BH4, Our findings that auranofin inhibited the metabolism of BH2 to BH4 in HaCaT cells provide further support for the idea that the drug inhibits DHFR (Fig. 8). This activity was time-dependent with a maximal inhibition after 2 h with 20 μM Auranofin. The IC50 for inhibition of DHFR in HaCaT cells by auranofin was 6.3 μM.

We next compared the activity of auranofin on SPR and DHFR activity in different human and rodent cell lines including Jurkat, BeWo HaCaT, SKNMC and CX-1, and PC12 and RAW264.7, respectively. Each of these cell lines was found to be responsive to auranofin (Table 1). With respect to inhibition of SPR, PC12 cells were the most sensitive to the drug (IC50 = 1.0 μM) followed by Jurkat (IC50 = 7.2 μM), BeWo (IC50 = 8.9 μM), HaCaT (IC50 = 10.7 μM), SKNMC (IC50 = 17.7 μM), RAW264.7 (IC50 = 21.3 μM) and CX-1 cells (IC50 = 25.1 μM). In general, DHFR was more sensitive to auranofin than SPR. This was most evident in BeWo cells (IC50 = 3.9 μM), HaCaT cells (IC50 = 6.3 μM) and CX-1 cells (IC50 = 7.3 μM).

### 3.2. Effects of auranofin on BH4-dependent enzyme activity

**Fig. 9.**
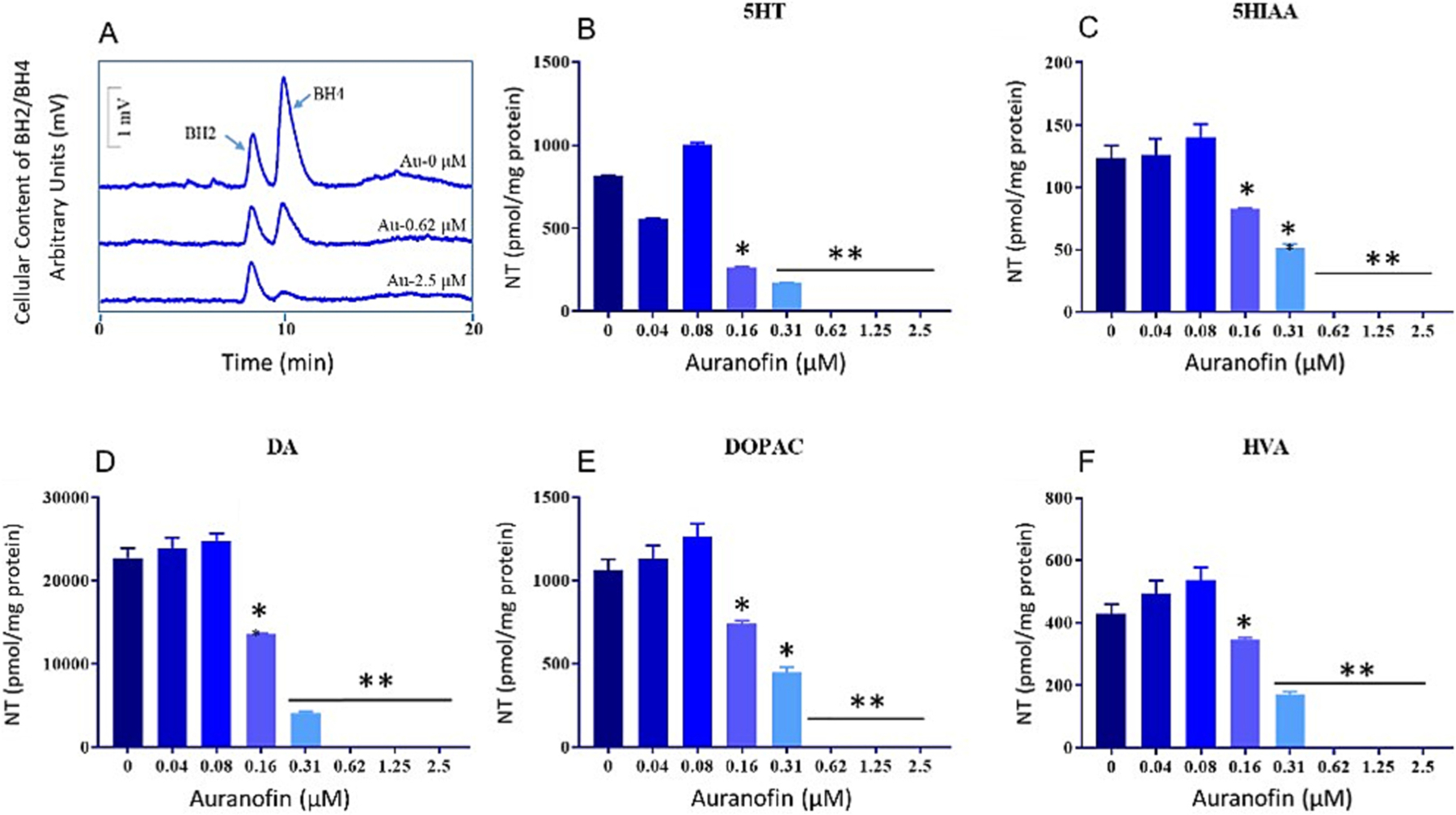
Effects of auranofin on the production of catecholamine and monoamine neurotransmitters by PC12 cells. Cells (8 × 10^6^/10-cm dish) were treated with control medium or medium containing increasing concentrations of auranofin for 21 h and then with sepiapterin (100 μM) for 3 h. Cells were analyzed for BH2 and BH4 as described in the Materials and Methods. *Panel A*. Top tracing, control; middle tracing, 0.62 μM auranofin; bottom tracing, 2.5 μM auranofin. *Panels B*–*F*. Cells treated with increasing concentrations of auranofin. After 8 h, catecholamine/monoamine neurotransmitters (NT) or metabolites (5-HT, 5-hydroxytryptamine; 5-HIAA, 5-hydroxyindoleacetic acid; DA, dopamine; DOPAC, 3,4-dihydroxyphenylacetic acid; HVA, homovanillic acid) were analyzed by HPLC with electrochemical detection. Bars, mean ± SE (n = 3). Significantly different (*p < 0.05, **p < 0.01) from control.

BH4 is a critical enzyme cofactor required to generate autocrine and paracrine monoamines via aromatic amino acid hydroxylases (Werner et al., 2011). These include serotonin, a neurotransmitter generated via tryptophan hydroxylase, dopamine, norepinephrine and epinephrine, formed from L-DOPA via tyrosine hydroxylase, and tyrosine via phenylalanine hydroxylase. Using PC12 cells, a neuroectodermal-derived rat cell line known to generate BH4 and aromatic amino acid hydroxylases required to generate catecholamines and monoamine neurotransmitters (Yang et al., 2015), we next determined if auranofin-induced inhibition of BH4 biosynthesis suppressed BH4-dependent aromatic amino acid hydroxylase activity. PC12 cells were found to generate serotonin and dopamine, as well as their metabolites, 5-hydroxyindoleacetic acid (5HIAA) and 3,4-dihydroxy-phenylacetic acid (DOPAC) and homovanillic acid (HVA), respectively (Fig. 9). Auranofin caused a concentration-dependent inhibition of the production of the monoamines and their metabolites (Fig. 9). These data indicate that BH4 is required for the activity of aromatic amino acid biosynthesis in PC12 cells.

## 4. Discussion

The therapeutic potential of gold salts, such as auranofin, has long been recognized due to their potent anti-inflammatory properties. Auranofin’s ability to slow the progression of rheumatoid arthritis marked a significant milestone in its development as a treatment for the disease (Finkelstein et al., 1976; Kean et al., 1997). Given auranofin’s high affinity for cysteine and selenocysteine, it is not unexpected that thioredoxin reductase- an enzyme with an accessible cysteine-selenocysteine pair in its active site- was among the first identified molecular targets of the drug (Gromer et al., 1998; Bindoli et al., 2009; Gencheva et al., 2022). By inhibiting the thioredoxin system, auranofin disrupts cellular redox homeostasis, leading to elevated oxidative stress. Beyond redox regulation, auranofin has also been shown to interact with components of the ubiquitin–proteasome system, which plays a critical role in maintaining protein homeostasis (Liu et al., 2014; Yan et al., 2023). One study demonstrated that auranofin inhibits proteasome-associated deubiquitinases (Liu et al., 2014), while another reported that it binds to and enhances the activity of ubiquitin-activating enzyme E1 (UBA1), which is important in initiation of protein ubiquitination (Yan et al., 2023). These findings suggest that the mechanism of action of auranofin may vary depending on drug dose and/or target cell type. For therapeutic applications in inflammatory diseases or cancer, engagement of multiple molecular targets may be necessary to achieve optimal efficacy.

Our findings identify a novel site of action for auranofin, namely the biosynthesis of BH4, a critical cofactor for the amino acid hydroxylases, nitric oxide synthases and alkylglycerol monooxygenase (Werner et al., 2011). In multiple cell lines, auranofin inhibited two successive steps in the BH4 biosynthetic pathway, SPR and DHFR, thereby blocking the formation of BH2 and BH4, respectively. These inhibitory effects were confirmed using recombinant SPR and DHFR. In cell-based assays, methotrexate, a well characterized inhibitor of DHFR (Sehrawat et al., 2023), blocked the conversion of BH2 from BH4. The treatment of cells with sepiapterin in the presence of methotrexate resulted in intracellular accumulation of BH2, which enabled the assessment of SPR activity. Conversely, treatment of cells with BH2, which is metabolized to BH4, allowed for evaluation of DHFR activity. However, it is important to note that BH4 can be further metabolized to BH2 and other derivatives (Sugiyama et al., 2009; Werner et al., 2011). BH2 can be recycled to BH4, however, not all metabolites are recyclable, and thus, our assay provides only an approximate measure of DHFR activity in cells (Sugiyama et al., 2009; Crabtree and Channon, 2011). Auranofin inhibited SPR and DHFR with similar potency across most cell types. PC12 cells were the most sensitive to auranofin (IC50 = 1.0 and 0.87 μM for SPR and DHFR, respectively), while Jurkat, BeWo, HaCaT, SKNMC, RAW264.7 and CX-1 cells were less sensitive to the drug, with IC50 values ranging from 7.2 to 25.1 μM for SPR activity and 3.9 to 17.2 μM for DHFR activity. Notably, in BeWo and CX-1 cells, DHFR activity was more sensitive to auranofin (IC50 = 3.9 μM and 7.3 μM, respectively), when compared to SPR activity (IC50 = 8.9 μM and 25.1 μM, respectively). The observed variability in sensitivity among cell types may reflect differences in intracellular drug accumulation and/or expression levels and biochemical properties of SPR and DHFR. Similar variability has been reported in the context of antifolate resistance in cancer chemotherapy, where altered DHFR expression and binding characteristics contribute to differential drug responses (Zhao and Goldman, 2003).

Our studies further demonstrate that auranofin effectively inhibited SPR-mediated redox cycling of menadione, a redox active quinone. These studies are consistent with our earlier work showing that SPR catalyzes a secondary adventitious reaction, chemical redox cycling; this reaction was inhibited by sulfanamide drugs, which also inhibit SPR-mediated reduction of sepiapterin (Werner et al., 2011; Yang et al., 2015). In the redox cycling process, SPR facilitates the one-electron reduction of redox-active compounds, including catechols derived from endogenous estrogens and quinones. The resulting semiquinone radicals rapidly react with molecular oxygen generating reactive oxygen species (ROS), which contribute to oxidative stress and inflammation (Dicker and Cederbaum, 1991; Rashba-Step and Cederbaum, 1994). Importantly, redox cycling of catechol estrogens has been implicated in mutagenesis and DNA damage, processes that may contribute to tumor initiation and progression (Cavalieri et al., 2000; Bolton and Thatcher, 2008; Fussell et al., 2011). The ability of auranofin to inhibit redox cycling suggests a potential mechanism by which the drug mitigates ROS-mediated toxicity. This property may contribute to its observed anti-inflammatory and anti-tumor effects, particularly in contexts where redox-active metabolites play a pathogenic role.

To assess the functional consequences of BH4 depletion, we used PC12 cells to examine the impact of auranofin on BH4-dependent enzyme activity (Yang et al., 2015). Auranofin treatment significantly reduced the biosynthesis of monoamines and catecholamines by inhibiting the activity of tryptophan hydroxylase and tyrosine hydroxylase, enzymes essential to produce serotonin, and dopamine, norepinephrine and epinephrine, respectively. These findings demonstrate that auranofin-mediated inhibition of BH4 synthesis can directly impair enzymes dependent on BH4 and neurotransmitter synthesis (Xiao et al., 2023).

Many cancer types, including pheochromocytoma (from which PC12 cells are derived), nerve sheath tumors, and certain breast and colon cancers, either produce or respond to monoamines and catecholamines (Kannen et al., 2020; Balakrishna et al., 2021; Ahangari and Norioun, 2025). These bioactive amines contribute to tumor progression by supporting cancer stem cell maintenance, promoting an undifferentiated cellular state, and regulating angiogenesis—an essential process for tumor growth (Flierl et al., 2008; Chakroborty et al., 2020; Kannen et al., 2020; Balakrishna et al., 2021; Ahangari and Norioun, 2025). These findings suggest that inhibition of monoamine and catecholamine synthesis provides a mechanism by which auranofin may have therapeutic implications in oncology. Other BH4-dependent enzymes such as nitric oxide synthases (NOS) and alkylglycerol monooxygenase have also been implicated in the pathogenesis of rheumatoid arthritis and cancer [35, 36]. In this regard, auranofin has been shown to suppress nitric oxide production in various cell types, including macrophages (Yamashita et al., 1997) and microglial cells (Madeira et al., 2015). It should be noted that BH4 depletion in cells leads to NOS uncoupling, a phenomenon that shifts NOS activity from nitric oxide production to superoxide generation, thereby contributing to oxidative stress (Castro et al., 2025). This mechanism may further enhance the pro-oxidant effects of auranofin in treated cells.

Both auranofin and methotrexate are used to treat rheumatoid arthritis (Cronstein and Sitkovsky, 2017). By inhibiting DHFR, these drugs act as folate antagonists and interfere with one carbon metabolism, which can suppress proliferation. Depending on the dose, other sites of action of methotrexate have been identified, notably adenosine signaling, which is known to contribute to its anti-inflammatory effects by activating adenosine receptors and suppressing inflammatory mediators (Cronstein and Sitkovsky, 2017; Zhao et al., 2022). Methotrexate has been reported to increase adenosine levels; activation of adenosine receptors on target cells initiates intracellular signaling pathways that antagonize inflammation (Cronstein and Sitkovsky, 2017; Pasquini et al., 2021). This process can result in suppression of key inflammatory mediators that contribute to inflammatory diseases including lipids, growth factors, cytokines and chemokines. The role of adenosine signaling in mediating the anti-inflammatory effects of auranofin remain to be investigated.

Sulfa drugs such as sulfasalazine are also known to inhibit SPR (Yang et al., 2015). These drugs are often used to treat inflammatory joint diseases reducing pain, inflammation and swelling (Guo et al., 2018; Khan et al., 2023). They have also been reported to slow the progression of joint damage (Guo et al., 2018). At the present time, it is unclear as to whether drugs such as sulfasalazine demonstrate therapeutic efficacy as a consequence of their effects on tetrahydrobiopterin metabolism.

Auranofin, a gold(I)-containing compound, exhibits a strong preference for binding to soft nucleophilic amino acids including cysteine, selenocysteine, lysine and histidine (Dos Santos, 2014). Formation of coordinated adducts, particularly in the catalytic pocket of an enzyme, can lead to inactivation. Earlier studies have shown that auranofin inhibits the thioredoxin (Trx) system by directly targeting the active site of thioredoxin reductase (TrxR) through coordination to the selenocysteine and by forming a coordinate Cys-Au-Cys bridge between a pair of cysteine residues in the active site of Trx further disrupting redox regulation (Quadros Barse et al., 2025). Beyond the Trx system, auranofin and its analogs have been found to inhibit other enzymes by interacting with catalytic lysine residues, such as those in cyclophilin and trypanothione reductase; these interactions contribute to the compound’s broad inhibitory profile (Zou et al., 2000; Ilari et al., 2012). Auranofin also modulates the ubiquitin–proteasome system by inhibiting proteasome-associated deubiquitinases, and enhancing the activity of UBA1, a ubiquitin-activating enzyme (Liu et al., 2014). In UBA1, gold (I) coordinates with Cys1039 in the ubiquitin fold domain, while the triethylphosphine ligand of auranofin is thought to interact with glutamic acid residues (E1037 and E1049) in the fold domain, promoting stronger interactions with E2 ubiquitin-conjugating enzymes (Yan et al., 2023).

Both human SPR and DHFR are NADPH-dependent enzymes that contain multiple lysine residues within their cofactor-binding domains (Schnell et al., 2004; Yang et al., 2013; Sehrawat et al., 2023). Notably, Lys-174 in human SPR and Lys-54 in human DHFR, are critical for proper NADPH positioning. These residues form bifurcated hydrogen bonds with the 2′-hydroxyl and 3′-hydroxyl groups of the ribose moiety of NADPH stabilizing its orientation within the active site (Schnell et al., 2004; Yang et al., 2013). We previously demonstrated that mutation of Lys-174 in SPR impairs its ability to catalyze the reduction of sepiapterin, underscoring the functional importance of this residue (Yang et al., 2013). Given the essential role of these lysine residues in cofactor binding, the NADPH-binding domain may represent a potential target site for auranofin in both SPR and DHFR; structural analysis has revealed other potential auranofin targets. In SPR, Cys-171 is located near the catalytic pocket, while in DHFR, Lys-68 plays a key role in substrate binding (Schnell et al., 2004; Yang et al., 2013). Both amino acid residues are also potential coordination sites for the gold(I) center of auranofin.

It should be noted that our studies focused on recombinant enzymes, cell lysates, and intact cultured cells. Whether auranofin also targets BH_2_ and BH_4_ metabolism in tissues relevant to rheumatoid arthritis or cancer remains to be determined. These additional studies would provide important information on the *in vivo* site of action of auranofin and help clarify its therapeutic mechanism. Our findings showing that SPR and DHFR are biochemical targets for auranofin is an important advance in understanding the pharmacology of this gold-based compound. As key enzymes in the BH4 biosynthetic and recycling pathways, SPR and DHFR are essential for maintaining cellular redox balance, neurotransmitter biosynthesis, lipid metabolism and nitric oxide production. Inhibition of these enzymes by auranofin has important biochemical and physiological effects that may contribute to its anti-inflammatory and antineoplastic activities.

In summary, our data demonstrates that auranofin inhibits two successive steps in the biosynthesis of BH4, the conversion of sepiapterin to BH2 by SPR and the subsequent reduction of BH2 to BH4 by DHFR. BH4 is an essential cofactor for key enzymes involved in inflammation, autoimmunity, and cell growth and development including aromatic amino acid hydroxylases, nitric oxide synthases and AGMO (Werner et al., 2011). Inhibiting BH4 biosynthesis may therefore contribute to auranofin’s therapeutic effects in diseases such as rheumatoid arthritis and cancer, potentially acting synergistically with its known targets in the thioredoxin system and the ubiquitin–proteasome system (Gromer et al., 1998; Bindoli et al., 2009; Liu et al., 2014; Gencheva and Arner, 2022; Gencheva et al., 2022; Yan et al., 2023; Quadros Barse et al., 2025). It should be noted that auranofin has been investigated as a treatment for infectious diseases caused by bacteria, fungi and parasites (Coscione et al., 2024; Chen et al., 2025). As many of these pathogens possess the ability to synthesize and utilize BH4, this pathway may also represent a therapeutic target in infectious diseases. Further investigation into the role of BH4 in mediating auranofin’s effects in inflammatory and infectious diseases and in cancer will be important in the development of more effective and targeted therapies.

## Figures and Tables

**Table 1:** Cellular inhibition of SPR and DHFR by auranofin.

Cells	Origin	Species	SPR^1^ IC50, μM	DHFR IC50, μM
PC12	pheochromocytoma	rat	1.0 ± 0.11	0.87 ± 0.09
Jurkat	leukemia	human	7.2 ± 0.17	5.4 ± 0.07
BeWo	choriocarcinoma	human	8.9 ± 0.64	3.9 ± 0.15
HaCaT	keratinocytes	human	10.7 ± 0.90	6.3 ± 0.75
SKNMC	neuroblastoma	human	17.7 ± 1.54	15.2 ± 0.53
RAW264.7	macrophage	mouse	21.3 ± 1.58	17.2 ± 0.63
CX-1	colon adenocarcinoma	human	25.1 ± 1.03	7.3 ± 0.10

1 Cells were treated with increasing concentrations of auranofin as described in the Materials and Methods. For SPR activity, BH2 formation from sepiapterin was assayed in the presence of methotrexate to inhibit further metabolism to BH4 through DHFR. For DHFR activity, BH4 formation from BH2 was assayed.

## Data Availability

Data will be made available on request.
